# Human papillomavirus 16-specific cell-mediated immunity in children born to mothers with incident cervical intraepithelial neoplasia (CIN) and to those constantly HPV negative

**DOI:** 10.1186/s12967-015-0733-4

**Published:** 2015-11-25

**Authors:** Hanna-Mari Koskimaa, Anna Paaso, Marij J. P. Welters, Seija Grénman, Kari Syrjänen, Sjoerd H. van der Burg, Stina Syrjänen

**Affiliations:** Medicity Research Laboratory and Department of Oral Pathology, Faculty of Medicine, Institute of Dentistry, University of Turku, Lemminkäisenkatu 2, 20540 Turku, Finland; Department of Obstetrics and Gynecology, Turku University Hospital, Turku, Finland; Department of Clinical Research, Biohit Oyj, Helsinki, Finland; Department of Clinical Oncology, Leiden University Medical Center, Leiden, The Netherlands

**Keywords:** Cell-mediated immunity, Children, Cytokine secretion, Human papillomavirus, Peripheral blood mononuclear cell, Serological antibody, T-cell immunity

## Abstract

**Objectives:**

HPV infections are detected in sexually naive children. This has raised the question about the role of early HPV infections in either protecting or predisposing to further HPV infections. HPV16-specific cell-mediated immunity (CMI) was studied in 10 case-children born to mothers with an incident cervical intraepithelial neoplasia (CIN) diagnosed during their 14-year follow-up (FU), and in 21 children born to mothers, who remained constantly HPV-negative (controls). The mean age of children was 12.3 years.

**Methods:**

Peripheral blood mononuclear cells were isolated from blood and stimulated with peptide pools covering HPV16 E2, E6 and E7. Proliferation of lymphocytes, their secretion of cytokines, and the frequency of regulatory T-cells were determined. The results were correlated with the HPV status and analyzed in a nested case–control setting.

**Results:**

All children, except two controls, displayed CMI against HPV16 E2, E6 and/or E7 peptides associated with type 1 and 2 cytokine secretion. Only two statistically significant differences were found in the nested case–control setting; (1) case-children had a higher TNF-α response to HPV16 E2 (p = 0.004) than controls and (2) controls had no response to HPV16 E7.2 peptide pool while 3/10 case-children had (p = 0.013). Totally, 50 and 57 % of the cases and controls, respectively, had HPV positive oral samples at some FU-visit. In addition, the children without any HPV antibodies before the age of 6 months showed proliferative responses of PBMC after HPV16 exposure more frequently than other children (p = 0.045).

**Conclusions:**

HPV16-specific CMI is common in young, sexually inexperienced children. This suggests that oral HPV infections occur frequently in children. Our results might also explain the previous findings that half of healthy adults demonstrate HPV-specific CMI irrespective of their partner/sexual status.

**Electronic supplementary material:**

The online version of this article (doi:10.1186/s12967-015-0733-4) contains supplementary material, which is available to authorized users.

## Background

Human papillomavirus (HPV) is the main cause of cervical cancer (CC) and also important in the etiology of other cancers, such as anal and head and neck cancers. HPV is also causing benign epithelial growths in mucosa i.e. papillomas and condylomas. The traditional concept of HPV infection as an exclusively sexually transmitted disease has strongly focused the research mainly to HPV infections in adults. However, the recent meta-analysis provided evidence that HPV infection affect also children under the age of sexual debut, as well as newborns at perinatal period [1]: HPV infection of the mother increases the risk of the newborn to acquire HPV infection by 33 % as compared to the newborns of HPV negative mothers. We have recently shown that oral HPV is prevalent in newborns (18 %). At delivery, mother-newborn pairs had similar HPV-genotype profiles, but this concordance disappeared in 2 months. Furthermore, the presence of HPV DNA in the placenta or in the umbilical cord blood increased the risk of oral HPV carriage of the newborn at delivery, and this association between placental HPV and oral HPV remained significant at least for the following 2 months [2]. These observations support the possibility of alternative routes of infection such as vertical transmission via infected placenta, cord blood, ascending cervical infection or infected birth canal, or horizontal transmission e.g. via breast-feeding, digital or oral to oral contacts [3–5].

All these data support the view that HPV infection can be acquired early in life. This has raised the question about the significance of early infections for later encounters with HPV in the view of immunity. It has been speculated that if a child acquires the first infection before the maturation of her/his immune system, the outcome of infection could eventually be either HPV-specific immunological tolerance or immune response with antibody production and cell-mediated immunity [1, 6–9]. However, because most studies on HPV immunity have still been focused on cervical HPV infections in women, the HPV-specific immunity in children has remained an unexplored area until our recent work [10]. Here, in our HPV family Study, we have studied HPV16-specific immunology in 10 mothers, who had developed cervical intraepithelial neoplasia (CIN) during the 14-year follow-up, and in their children. To our surprise, all 10 children had a T-cell response against HPV16 E2-, E6-, or E7 peptides, supporting the view that HPV infection has already been acquired at early age by non-sexual routes.

To further elucidate the dynamics of HPV-specific cell-mediated immunity (CMI) we compared the responses of these 10 children born to the mothers who developed incident CIN, to those 21 children whose mothers remained constantly HPV-negative at cervix during the 6 year follow-up. The mononuclear cells isolated from the venous blood of the children were stimulated with HPV16 peptide pools and the following CMI response was determined by measuring the proliferation of lymphocytes, their secretion of cytokines, and the frequency of regulatory T-cells.

## Methods

### Children

The Finnish HPV Family Study is a longitudinal cohort study conducted at the Department of Oral Pathology, Institute of Dentistry, University of Turku and the Department of Obstetrics and Gynecology, Turku University Central Hospital. The study plan was approved by the Research Ethics Committee of Turku University Hospital (#3/1998 and 45/180/2010). Originally, 329 pregnant women at their 3rd trimester of pregnancy and all their newborns (n = 331; includes two twins) were enrolled in the study between 1998 and 2001, as described previously [9, 11, 12]. As part of the Finnish Family HPV Study, two subgroups of the children were selected for this study; the first (case) group comprised 10 children, 3 girls and 7 boys, whose mothers developed an incident CIN during the follow up [13]. The second (control) group consisted of 21 children, 13 girls and 8 boys, whose mothers tested constantly negative for genital HPV infection during the follow-up (FU) of 6 years (mean 54.9 months, median 62.4 months) [14, 15]. The children were recalled for blood sampling and written informed consent was obtained from all these children and their parent. The mean age of all children studied was 12.3 years with a range of 10.3–14.6 years. None of these children had had any sexual contacts or received prophylactic HPV vaccination before the entry of this sub study.

The authors acknowledge the concept of the Minimal Information About T-cell Assays (MIATA) framework, which was recently published [16, 17]. Therefore, detailed information is provided as structured in the proposed 5 modules by MIATA: the sample, the assay, the data acquisition, the data analysis and the laboratory environment in which the human T-cell assays were performed.

### HPV DNA detection and HPV antibody screening

Oral scrapings from the children were taken at birth, at the age of 3 days, and 1, 2, 6, 12, 24, 36 and 72 months using cytobrush (MedScand) as described earlier [11]. DNA was extracted from scraping samples using high salt method [18, 19]. First the high-risk HPV types were detected by nested PCR with MY09/MY11 and GP05+/GP06+ primers. PCR products were run in 2.0 % agarose gel, and hybridized with a digoxigenin-labeled, high-risk HPV-oligoprobe cocktail (HPV-types 16, 18, 31, 33, 35, 39, 45, 51, 54, 56 and 58). HPV genotyping was done with Multimetrix^®^ assay (Multimetrix, Regensburg, Germany) as described earlier [20], which detects the following low-risk (LR)-HPV-types:6, 11, 42, 43, 44, 70 and high (HR)-HPV-types:16, 18, 26, 31, 33, 35, 39, 45, 51, 52, 53, 56, 58, 59, 66, 68, 73 and 82. The median fluorescence intensity (MFI) of at least 100 beads was computed for each bead set in the sample. The cut-off value for each HPV-probe individually was defined: 1.5× MFI + 5 MFI.

Blood samples for detection of serum antibodies for HPV were collected from children at the age of 1, 2, 6, 12, 24, and 36 months and sent for analysis to DKFZ, Heidelberg (Germany). Serum antibodies for the major capsid protein L1 of HPV types 6, 11, 16, 18 and 45 were analysed by multiplex HPV-serology, based on glutathione S-transferase fusion-protein capture on fluorescent beads. Sera were scored as positive when the antigen-specific MFI values were greater than the cut-off level of 200 or 400 MFI for L1 antigens of individual HPV-types [20].

### Blood samples for CMI studies

Venous blood samples were collected from children and the peripheral mononuclear cells (PBMCs) were isolated as described previously [10, 21, 22]. Briefly, samples of 54 mL of venous blood were drained from children into sodium-heparin collection tubes. The PBMCs were isolated by centrifugation over Ficoll-Hypaque gradient (GE Healthcare Life Sciences, Uppsala, Sweden). Of these PBMCs, ~13.5 × 10^6^ cells were used for determining the proliferative capacity of HPV16-specific T-cells by short-term lymphocyte stimulation test (LST). The remaining PBMCs were frozen in 80 % Fetal Bovine Serum (FBS, Biowest, EU quality) and 20 % DMSO (Merck, Darmstadt, Germany) and stored in liquid nitrogen. The serum from 9 mL blood collected into clotting tube was isolated by centrifugation for 7 min at 1800*g*. Obtained autologous serum was used for short-term T-cell proliferation assay.

### HPV16 peptides

Eight peptide pools of HPV16 peptides were used to determine the proliferative capacity of HPV16-specific T-cells, for cytokine polarization analysis and detection of the HPV16-specific Foxp3+ regulatory T-cells, as described previously [10, 21, 22]. Panels of overlapping 30-35 mer peptides with HPV16 E2, E6, and E7 protein sequences were synthesized by solid phase peptide synthesis (SPPS) method with >95 % purity (ChinaPeptides Co. Shanghai, China), with a 14 (for 30-mer) or 15 (for 35-mer) amino acid (aa) overlap. Two pools of E2 peptides (E2.1 and E2.2) consisted of 12 or 11 (30-mer) peptides, respectively. Four pools of E6 and two pools of E7 peptides (E6.1-E6.4 and E7.1 and E7.2) consisted of two 32-mer or 35-mer peptides, respectively [10]. The numbers of covered amino acids of each protein are shown in Additional file 1. Memory response mix (MRM) stock solution (50×), consisting of tetanus toxoid, 0.75 fL/mL (Statens Serum Institut, Copenhagen, Denmark), Tuberculin PPD, 5 µg/mL (Statens Serum Institut), and Candida albicans, 0.015 % (Greer Laboratories, Lenoir, USA) was used as a positive control for the proliferation assays and cytokine production capacity of the PBMCs.

### Determination of the proliferative capacity of HPV16-specific T-cells by short-term lymphocyte stimulation test (LST)

LST was performed as described previously [10, 23, 24]. Briefly, PBMCs were cultured in eight replicative wells for each peptide pool with a final concentration of 5 µg/mL per peptide. PBMCs cultured with MRM were used as a positive control and with no antigen (medium-only) as a background control. After culturing for 6 days, media from each well was collected for cytokine analysis. The proliferation of cells were determined by [^3^H]-Thymidine incorporation assay. The cut-off value for counts per minute (CPM) values was determined by the average plus 3× SD of the eight medium-only control wells. Stimulation index (SI) was calculated as the average of tested eight wells divided by the average of the medium-only control wells. The proliferative response was defined positive if the CPM values of at least six of the eight wells were above the cut-off value and if the SI was ≥3.

### Cytokine polarization analysis

The supernatants collected from eight replicative wells of LST at day 6 of culturing were pooled and analyzed using Cytometric Bead Array (CBA) human enhanced sensitivity flex set system (BD Biosciences, Temse, Belgium) according to the manufacturer’s instructions. In this array, the levels of IFN-γ, TNF-α, IL-2, IL-4, IL-5, IL-10, and IL-17A were determined, as previously described [10, 23]. The detection limits for the cytokines were based on standard curves complying with the limit of 274 fg/mL described by the manufacturer. The positive antigen-induced cytokine production was defined as a cytokine concentration >2× the concentration of the medium-only control.

### Identification of HPV16-specific CD4 + CD25 + Foxp3 + regulatory T-cells

Regulatory T-cell identification was performed as previously described [10, 21, 25]. Briefly, PBMCs were cultured with the peptide pools as in the LST. Cells cultured with MRM were used as a positive control and cells without antigen (medium-only) were used as a background control. After 7 days of culturing, the cells were harvested and stained with surface markers CD25 (1:25) (Anti-CD25 FITC, clone M-A251, BD Pharmingen, San Diego, CA, USA), CD4 (1:100) (Anti-CD4-APC, clone RPA-T4, BD Pharmingen), CD8 (1:30) (Anti-CD8 PerCP-Cy5.5; clone SK1, BD Pharmingen), and intracellular marker Foxp3 (PE anti-human FOXP3, clone 206D, Biolegend) or isotype control (PE Mouse IgG1, κ Isotype Ctrl, Biolegend). After staining the cells were measured by the flow cytometer BD FACSCalibur (BD Bioscience). Analysis was performed by using Flowing Software, version 2.5.0 (Cell Imaging Core, Turku Centre for Biotechnology, Turku, Finland). The fluorescent intensity of MRM-stimulated and medium-only control cells was used to set the gates for the other samples. An antigen-induced alteration in the population percentage was defined as a change of at least 2× the corresponding percentage in the medium-only controls.

### Statistical analysis

All statistical analyses were run using IBM SPSS^®^ (IBM, Inc., New York, USA) software package (IBM SPSS Statistics for Windows, version 22.0.0.1). Frequency tables were analysed using the χ^2^-test, with the likelihood ratio or Fisher’s exact test for categorical variables. Differences in the means of continuous variables were analysed using non-parametric (Mann–Whitney) tests for two independent samples. All statistical tests were two-sided and declared significant at *p* value ≤0.05.

### Laboratory environment

The laboratory of the Oral Pathology at the Institute of Dentistry, Faculty of Medicine, University of Turku, Turku, Finland, is a research laboratory where the T-cell assays are performed according to SOPs, including the predefined criteria for positive responses.

## Results

### Children’s oral HPV DNA status and HPV serology

All children of this study were followed from birth until the age of 6-years. HPV DNA status of the oral mucosa was determined at birth, at the age of 3 days, and 1, 2, 6, 12, 24, 36 and 72 months. HPV serology to genotypes 6, 11, 16, 18, and 45 was determined at same time points starting from the age of 1–36 months. Table 1 summarizes the number of children bearing oral HPV DNA and HPV antibodies at any visit during the FU. The additional tables show the FU data of each child in more detail (see Additional files 2 and 3). Altogether, over half of the children, 5 of the 10 case-children and 12 of the 21 controls, tested occasionally HPV positive in their oral samples during the FU. HPV16 was found in oral mucosa in 1/10 (ID8) and 7/21 children of the cases and the controls, respectively. 3 of the 7 HPV16 positive controls had also HPV16 antibodies (ID14, ID16, and ID19), but not at the same time points when they tested HPV16 DNA positive. Additional 3 case-children and 3 control-children had HPV16 antibodies remaining all the time HPV16 DNA negative. Two children in the control group (ID12 and ID22) had HPV16 DNA, but no HPV-specific antibodies for any of the tested HPV types. Overall, HPV antibodies were found in 9 children in the case group and in 16 children in control group. From these, 5 case-children and 11 control-children had HPV serology to the same HPV genotypes as their mothers at baseline (before delivery). These similar antibodies were in most cases (4/5 and 9/11) detectable in first 6 months of child’s life. Only 3 children; ID1 in the case group, ID24 and ID30 in the control group, remained HPV DNA and HPV seronegative during the FU.

**Table 1:** The number of children bearing oral HPV DNA and HPV antibodies at any visit during the FU

HPV genotype	Case group (n = 10)		Control group (n = 21)	
	Oral HPV DNA	HPV L1 antibodies	Oral HPV	HPV L1 antibodies
Any HPV type	5 (50 %)	9 (90 %)	12 (57 %)	16 (76 %)
HPV6	3	8	4	10
HPV11	0	0	1	7
HPV16	1	3	7	6
HPV18	2	2	2	8
HPV31	2		0	
HPV33	1		0	
HPV39	0		1	
HPV45	0	0	0	1
HPV70	1		1	

Oral HPV was tested at birth, at the age of 3 days, 1, 2, 6, 12, 24, 36 and 72 months. Serum antibodies for HPV capsid protein L1 were tested at the age of 1, 2, 6, 12, 24, and 36 months

### Short-term lymphocyte stimulation test

**Fig. 1:**
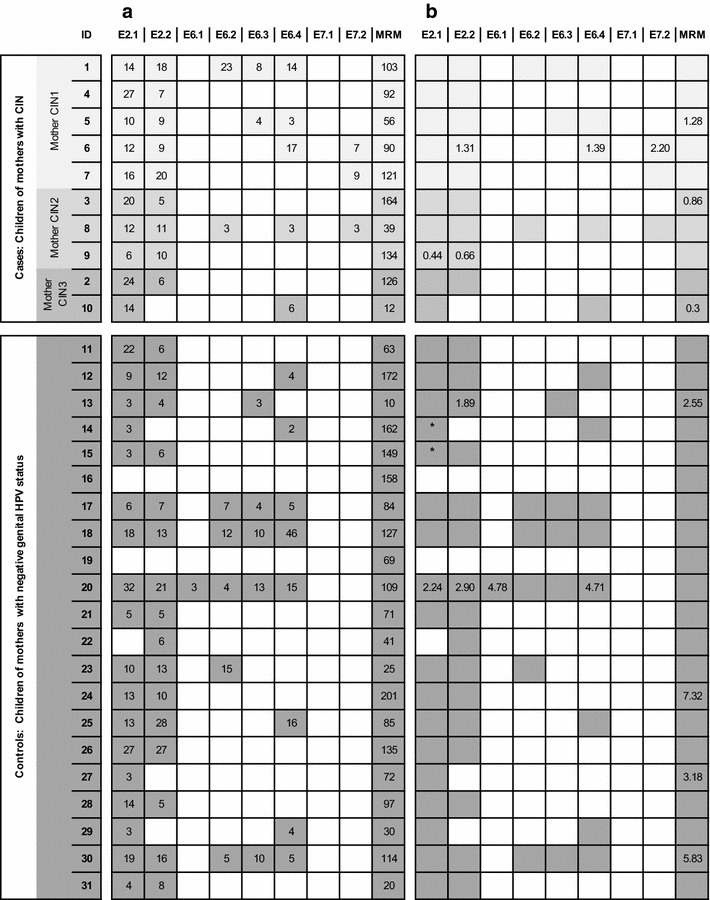
Results from LST and Foxp3 assay. **a** Results of HPV16-specific lymphocyte stimulation test (LST). Only positive responses are marked with *colored box* and stimulation indexes under the corresponding peptide pools. Memory response mix (MRM) was used as a positive control. **b** Percentages of CD4 + CD25 + Foxp3 + cells (Tregs). Only positive (upregulation of Tregs) responses are shown. *Colored box* indicates the coincidence positive response in LST test. Up-regulation of Tregs is defined as at least twice the percentages of Tregs in the medium only control. *No PBMCs were obtainable for this test

The HPV16-specific proliferative responses of PBMC in both groups are shown in Fig. 1a. PBMCs of all tested children in both groups showed proliferative response after MRM stimulation, indicating that the cells had the capability to recognize the common antigens and to proliferate as a response. The average SI against MRM was 95 in both groups although the range of SI was wide (12–164 in cases and 10–201 in controls) (see Additional file 1). HPV16-specific T-cell response against one or both of HPV16 E2 peptide pools was found in all 10 children of the case group and in 19 of 21 control-children. In the controls, only two children (ID16 and ID19) had no HPV16-specific T-cell response to any of the 8 peptide pools tested. An E6-specific response at least to one of the peptide pools of E6, was detected in approximately 50 % of all children (5/10 cases and 10/21 controls). Among the E6 peptides the peptide pool E6.4 was the most prevalent to stimulate PBMCs (5/10 cases and 8/21 controls). E6.2 and E6.3 induced T-cell responses in 2/10 cases and 5/21 controls. Only one child of the control group (ID20) had response against the pool E6.1. No children in the control group had reactivity against either peptide pools of E7, while three children of the case group had response against the pool E7.2. This was also the only statistically significant difference between the two groups in the LST test.

In addition to mother HPV and CIN status, the LST results of all 31 children of case and control groups were also analyzed according to the presence of maternal HPV antibodies in their sera as infants. The children were grouped accordingly: (1) children (n = 13) with HPV antibodies before the age of 6 months and (2) children without any detectable HPV antibodies before the age of 6 months. This grouping resulted in a statistically significant difference in the proliferative responses against HPV16 peptide pools. The children without any HPV antibodies before the age of 6 months showed proliferative responses of PBMC after HPV16 exposure more frequently than other children (p = 0.045).

### Cytokine polarization analysis

**Fig. 2:**
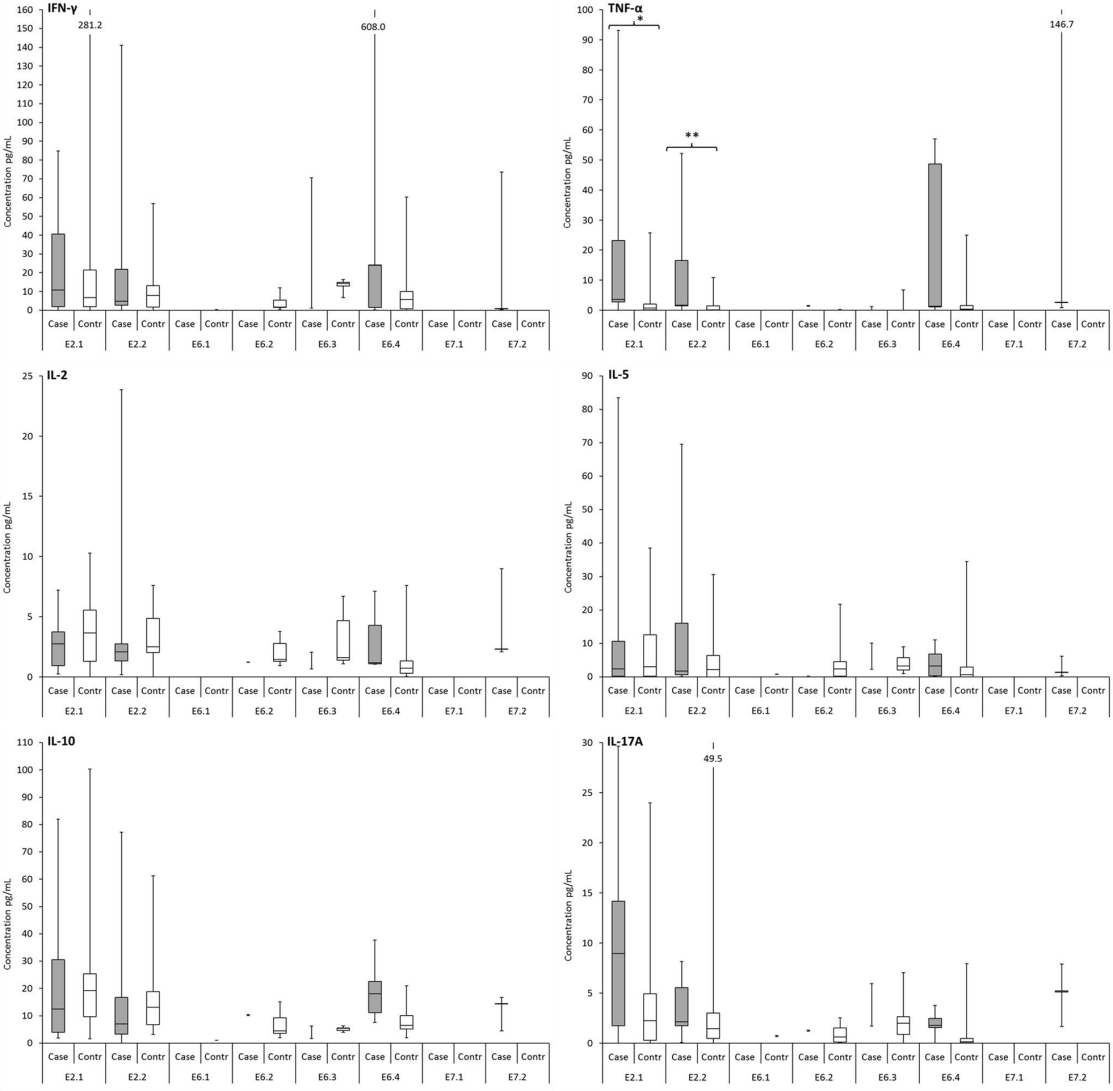
HPV16 E2, E6, and E7-specific cytokine secretion in case and control groups. Secreted cytokines were determined only from PBMCs showing proliferative response against HPV16 peptides (LST+). The *box* is bounded on the *top* by the third quartile, the *bottom* by the first quartile, and divided by the median. The minimum and maximum are indicated by the *whiskers*. If three or fewer values are detected the median and/or min and max values are connected with *vertical line*. The concentrations of TNF-α showed statistically significant difference between groups *p = 0.031, **p = 0.039

The cytokine levels of IFN-γ, TNF-α, IL-2, IL-4, IL-5, IL-10, and IL-17A after stimulation of PBMC with HPV16E2, E6 and E7 peptides are summarized in Fig. 2. Secreted cytokines were determined only from PBMCs showing proliferative response against HPV16 peptides (LST+). Generally, the levels of all cytokines were below 100 pg/mL, but the range was wide between maximum and minimum rates. IFN-γ and IL-17A were the most frequently detected cytokines among all children followed by IL-2, IL-5 and IL-10, although the levels of IL-2 were considerably low. IL-4 was detected only in two cases as a response only to MRM. TNF-α concentration was statistically significantly higher in the cases than in the controls after stimulation with HPV16 E2.1 and E2.2 peptide pools (P = 0.031 and P = 0.039, respectively). The secretion of IFN-γ was almost always, with the exception of two children in the control group (ID19 and ID31), accompanied with secretion of IL-5 (Th2 cytokine).

**Fig. 3:**
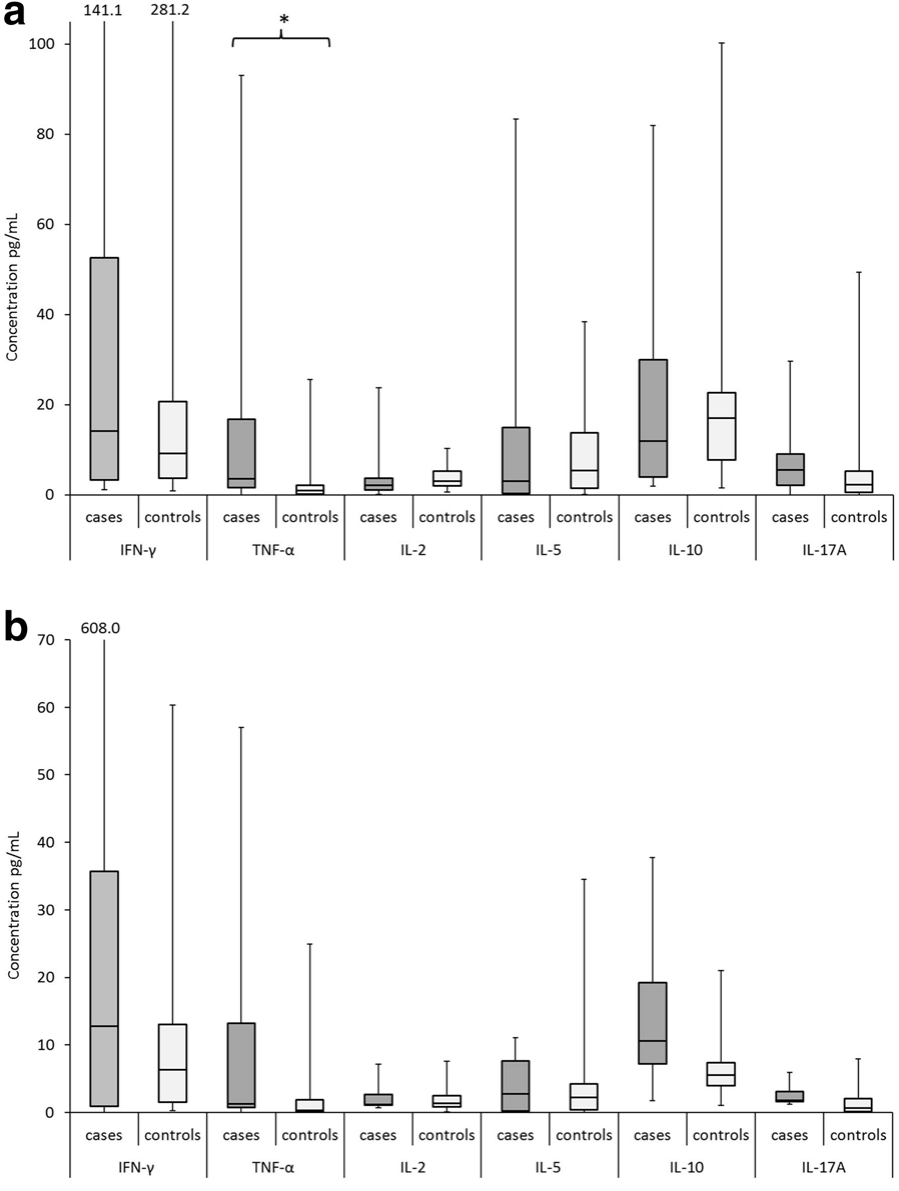
The cytokine levels secreted by PBMCs as response to stimulation with **a** HPV16 E2 and **b** E6 peptides. Cytokine concentrations were measured only from PBMCs with positive proliferative response (LST positive). The cytokines detected against pools of HPV16 E2 or E6 are combined. HPV16 E7 is not shown, as only three children from case group showed response against HPV16 E7. The *box* is bounded on the *top* by the third quartile, the *bottom* by the first quartile, and divided by the median. The minimum and maximum are indicated by the *whiskers*. If three or fewer values are detected the median and/or min and max values are connected with *vertical line*. The secretion of TNF-α as a response to HPV16 E2 peptides showed statistically significant difference between groups *p = 0.004

Figure 3 is presenting the cytokine concentrations according to the stimulation. Of the tested cytokines the concentrations of IFN-γ and IL-10 were highest in both groups. Also here the only difference in cytokine levels between TNF-α concentration was statistically significantly higher in the cases than in the controls when the effects of pooled E2 and E6 peptides were assessed.

### HPV-specific CD4 + CD25 + Foxp3 + regulatory T –cell detection

The frequency of CD4 + CD25 + Foxp3 + regulatory T-cells (Tregs) after 7-day stimulation of PBMCs was analyzed with flow cytometry (Fig. 1b). In the case group, two of the 10 case-children had HPV16-specific Foxp3+ Treg responses: child ID6 had responded to three peptide pools and ID9 had response against both pools of E2. Both had also a positive Treg response against MRM.

Two children (ID13 and ID20) in the control group had positive Treg responses against peptide pool E2.2., and child ID20 also against peptide pools E2.1, E6.1, E6.4, and E7.2. Child ID13 had also response against the MRM but not the child ID 20.

## Discussion

Since the cell-mediated immune response (CMI) has been regarded as the paramount player in the resolution of the HPV infection and diseases induced by HPV, the present HPV-specific T-cell memory of individual might provide information on both, the encountered infections and possible reactivity for infections to come. Recently, we published the first work showing that HPV-specific CMI exists already in children naïve to any sexual contacts or HPV vaccinations [10]. As all mothers of these 10 children had developed an incident CIN during the 14-year follow-up, we wanted to further investigate if the mother’s disease caused by HPV had effect on the HPV-specific immune response in her child. Our hypothesis was that the children whose mothers tested HPV negative would not display any HPV-specific immunity. Therefore, we designed the present study as a continuum for our previous study. Here, we investigated further the HPV16-specific T-cell responses in 21 children born to mothers, who tested HPV negative in their cervical samples at every visit during the 6-year FU. We treated the acquired results of HPV16-specific immune response in both groups, the 21 children of present and the 10 children of previous work, in nested case–control setting. As previously, we used PBMCs isolated from venous blood of these children which were stimulated by HPV16 peptide pools. The proliferation of lymphocytes, their secretion of cytokines, and the frequency of regulatory T-cells were determined, and compared in a case–control setting. Surprisingly, the responses of the 21 children in the control group showed similarity to our previous results of the 10 children in the case group.

Altogether, proliferative responses against HPV16 E2 peptides were the most predominant in both groups; all 10 children of the case group and 18 of the 21 children of the control group displayed E2-specific immunity, especially against the peptide pool E2.1. (amino acids 1–195 of HPV16 E2 protein). Only one child in the control group reacted only with the peptide pool E2.2. but not with pool E2.1.

The HPV16 E6 peptides induced proliferative responses in 5 of 10 children in case group and 10 of 21 children in control group. These observations are congruent with several studies, where the HPV16 E2 and E6-specific immune responses are prevalent among healthy adults [23, 24, 26]. The results showed statistically significant difference between the two groups only regarding the responses against HPV16 E7.2 peptide pool, where no response was seen in the control group. This unresponsiveness to HPV16 E7 peptide pools is expected in healthy adult subjects [24, 27, 28], but no earlier data exist on children.

As HPV E2 is abundant in infected cells at the stage of viral amplification, the reactivity to HPV16 E2 peptides may be a sign of a previous replicative HPV16 infection [29]. This raises the question about possible cross-reactivity of T-cells that were initially mounted to infections with other HPV types [23, 24]. Previous studies showed that cross-reactive HPV16 E2- and E6-specific T-cells are rarely found, indicating that also children in the control group carry true circulating HPV16-specific memory T-cells in their system [23, 30, 31]. Importantly, several children displayed positive HPV16 DNA tests and/or HPV 16 seropositivity including also the children born to mothers constantly HPV negative during follow-up. Moreover, the follow-up of these children shows that over half of the children, both the cases and the controls, have had detectable HPV DNA of any type in their oral samples. Also in our previous work, oral HPV DNA was found in 17.9 % of 331 newborns already by the age of 2 months [2]. These observations together suggest that the oral mucosa is a site for primary HPV infection that usually go unnoticed and serves as a site for the induction of HPV-specific humoral and cellular immune responses, the memory cells which are still detected later in life [23, 24]. As our previous studies imply that the mother is the most likely transmitter of HPV to her newborn [2, 9, 11], the detection of HPV16 DNA and antibodies in children of HPV negative mothers raises the question about the source of HPV infection in these children. This can be explained by possible sampling errors or latent HPV infection undetectable leading to a false HPV negative result of the mother: HPV DNA detection is based on the collected cells. Since asymptomatic infections are not visible, the collected sample might not contain enough HPV infected cells and/or the copy numbers in the infected cells are too low for HPV DNA detection. Also the other HPV transmission routes apart of the mother should be taken into consideration. These include nosocomial infection or infection from siblings or other family members via kissing or through hands. Alternatively, the relatively high HPV detection rates in children might partly be explained by the possibility of false positive HPV results as an effect of passenger or transient HPV rather than true infection. The HPV testing by Multimetrix method utilizing nested PCR products is improbable source of errors, since it is well-defined as sensitive and specific method controlled by simultaneous analysis of several HPV negative control samples between the study samples (every 8th sample is a negative control).

The relatively high HPV18 seropositivity found among the control-children is surprising as the prevalence of oral HPV18 is low. Furthermore, HPV 18 genotype has a strong association with cancer or cancer development. The levels of HPV18 L1antibodies in the sera of were low, but still exceeding the cut-off value of 200MFI. Only two children (ID 182 and 228) had titers higher than the 600MFI. The HPV serology was analyzed in the reference laboratory (DKFZ, Heidelberg, Germany).

In the present study, the HPV16-specific proliferative T-cells secreted higher levels of cytokines than the cells with no detectable proliferation response. The levels of cytokine TNF-α showed difference between the two groups in that the children in the case group had higher levels. Altogether, the T helper type 1 cytokine IFN-γ and T helper type 2 cytokine IL-5 secretion, characteristic to the HPV16-specific immune response in healthy population [22], were frequently seen among children of this study, as well.

Furthermore, we detected Foxp3+ T regulatory-cell activation in four children. Two children in the case group and two children in the control group had Foxp3+ Treg responses against peptide pools of HPV16 E2. In addition, two of them also reacted against peptide pools of E6 and, one against peptide pool E7.2. Since Foxp3+ T regulatory-cells are known to suppress immune responses and several studies suggest that association between increased CD4+ Foxp3+ frequency and persistent HPV infection and/or progression of cervical dysplasia and other malignancies [32–35], our observations rouses a question about the significance of Tregs in healthy children and the bearing it might have for later HPV infections.

## Conclusions

To conclude, the HPV16-specific immunity seems to be common in young, sexually inexperienced children. Though, the HPV infection status or HPV related disease of the mother did not show any effect on the HPV-specific immunity of her child. Our data also supports the view that in many cases, the first HPV infection is acquired at early infancy, before the onset of sexual life. In fact, these results show that oral HPV infections occur much more frequently in young children than expected before. The HPV-specific humoral and cellular immunity induced by these early infections could explain previous observations showing T-cell immunity in about half of healthy adults irrespective of their partner/sexual status. Even though the number of the study subjects was limited, the present well characteristic follow- up cohort is unique in its design, and gives a strong evidence of the existence of HPV immunity in children. However, the higher number of study subjects would be needed for the discrimination of the possible effects of the mother’s HPV status or special characteristics of the child to the HPV immunity.

These data may also give rise to the discussion about the significance of these early HPV infections to the effectiveness of prophylactic HPV vaccines. Thus, the virus like particles in HPV vaccines are shown to induce neutralizing antibodies against HPV L1 remarkably more efficiently than natural infections and be very efficacious in reduction of infections by vaccine related HPV types [36–38]. However, one possible scenario could be, that the children bearing HPV DNA and/or HPV-specific T-cells, but no HPV antibodies, might have some systemic mechanism to impair or even prevent also the seroconversion induced by HPV vaccine.

Another issue to be resolved is that despite the protection resulting from natural infection and/or HPV vaccination, the sexual behavior still remains a risk factor for cervical cancer development [39]. A possible explanation could be that the systemic immunity is not sufficient for prevention of the infection, if there is a lack of a real local immunity in the cervix.

## Additional files

10.1186/s12967-015-0733-4 Proliferative T-cell responses against peptide pools of HPV16 E2, E6 and E7 of 31 children. The box is bounded on the top by the third quartile, the bottom by the first quartile, and divided by the median. The minimum and maximum are indicated by the whiskers. If three or fewer values are detected the median and/or min and max values are connected with vertical line.
10.1186/s12967-015-0733-4 The oral HPV and HPV antibodies of the case-children from the baseline to 6 years (72 months). Oral HPV was tested at every time point, serum antibodies at the age of 1, 2, 6, 12, 24, and 36 months. Number in the box indicates the detected HPV type. Negative result is indicated by x and no sample by empty bar.
10.1186/s12967-015-0733-4 The oral HPV and HPV antibodies of the control-children from the baseline to 6 years (72 months). Oral HPV was tested at every time point, serum antibodies at the age of 1, 2, 6, 12, 24, and 36 months. Number in the box indicates the detected HPV type. Negative result is indicated by x and no sample by empty bar.

## Abbreviations

CBA: cytometric bead array
CC: cervical cancer
CIN: cervical intraepithelial neoplasia
CMI: cell-mediated immune
CPM: counts per minute
Foxp3: forkhead box P3
FU: follow up
HPV: human papillomavirus
HR: high-risk
LST: lymphocyte stimulation test
MRM: memory response mix
SI: Stimulation index
SPPS: solid phase peptide synthesis
