# Supplementary material for: Human papillomavirus 16-specific cell-mediated immunity in children born to mothers with incident cervical intraepithelial neoplasia (CIN) and to those constantly HPV negative
Source: J Transl Med. 2015 Nov 25;13:370. doi: 10.1186/s12967-015-0733-4 (PMC4659171; doi:10.1186/s12967-015-0733-4)
Supplement: Supplementary file 1 — 10.1186/s12967-015-0733-4 Proliferative T-cell responses against peptide pools of HPV16 E2, E6 and E7 of 31 children. The box is bounded on the top by the third quartile, the bottom by the first quartile, and divided by the median. The minimum and maximum are indicated by the whiskers. If three or fewer values are detected the median and/or min and max values are connected with vertical line. [file 12967_2015_733_MOESM3_ESM.pdf]

		baseline	3 d	1 mo	2 mo	6 mo	12 mo	24 mo	36 mo	72 mo
ID11	oral HPV	x				x	x	x	x	x
	Antibodies			11		x	x	x	x	
ID12	oral HPV	16, 39	16	x	x	x				39
	Antibodies			x						
ID13	oral HPV	x	x	x	x	x	x	x	x	x
	Antibodies			11, 16	11, 16	x	x	x	x	
ID14	oral HPV	x	x	x	x	x	x	x	16	x
	Antibodies				6, 16, 18	6, 18	6, 18			
ID15	oral HPV	x	x	x	x		x			
	Antibodies			6	6		16, 45			
ID16	oral HPV	x	16	x	11	x	x	x	x	
	Antibodies			x	6, 11	6, 11	6, 11	16, 18	6	
ID17	oral HPV	6	x	6	x	x	x	x	x	
	Antibodies				x	x	x	x	18	
ID18	oral HPV	x	x	x	x	x	x	x	x	
	Antibodies			18	18	6, 18	6	6	x	
ID19	oral HPV	x	x	16	x	x	16	x	x	x
	Antibodies			11, 16	16	x	x	6, 11	6	
ID20	oral HPV	x	x	x	x	18	70	x	x	
	Antibodies			x	x	x	x	6,11	6	
ID21	oral HPV	x	x	x	x	x	x	x	6	
	Antibodies			x	x	6, 18	6, 18			
ID22	oral HPV	x	x	x	x	16	16	x	x	
	Antibodies			x	x	x				
ID23	oral HPV	x	16	x	x	x	x	x	x	
	Antibodies			x	x	x	x	x	18	
ID24	oral HPV	x	x	x	x	x	x	x	x	
	Antibodies			x		x	x	x	x	
ID25	oral HPV	6	x	x	x	x			x	
	Antibodies			18	x	x	x	6, 11	6	
ID26	oral HPV	18	x	16	x	x			16	x
	Antibodies			x	x	x	x	x	x	
ID27	oral HPV	x	x	x	x	x				x
	Antibodies			x	x	x	x	18		
ID28	oral HPV	x	x	x	x	x	x			
	Antibodies			x			6, 11			
ID29	oral HPV	x	x	x	x	x	x	x	x	x
	Antibodies			6	6	x		x	x	
ID30	oral HPV	x	x	x	x	x	x	x	x	x
	Antibodies			x	x	x	x	x	x	
ID31	oral HPV	x	6	x	x	x	x			
	Antibodies			16	x	x	x			
