# Supplementary figures and images for: Human papillomavirus 16-specific cell-mediated immunity in children born to mothers with incident cervical intraepithelial neoplasia (CIN) and to those constantly HPV negative
Source: J Transl Med. 2015 Nov 25;13:370. doi: 10.1186/s12967-015-0733-4 (PMC4659171; doi:10.1186/s12967-015-0733-4)

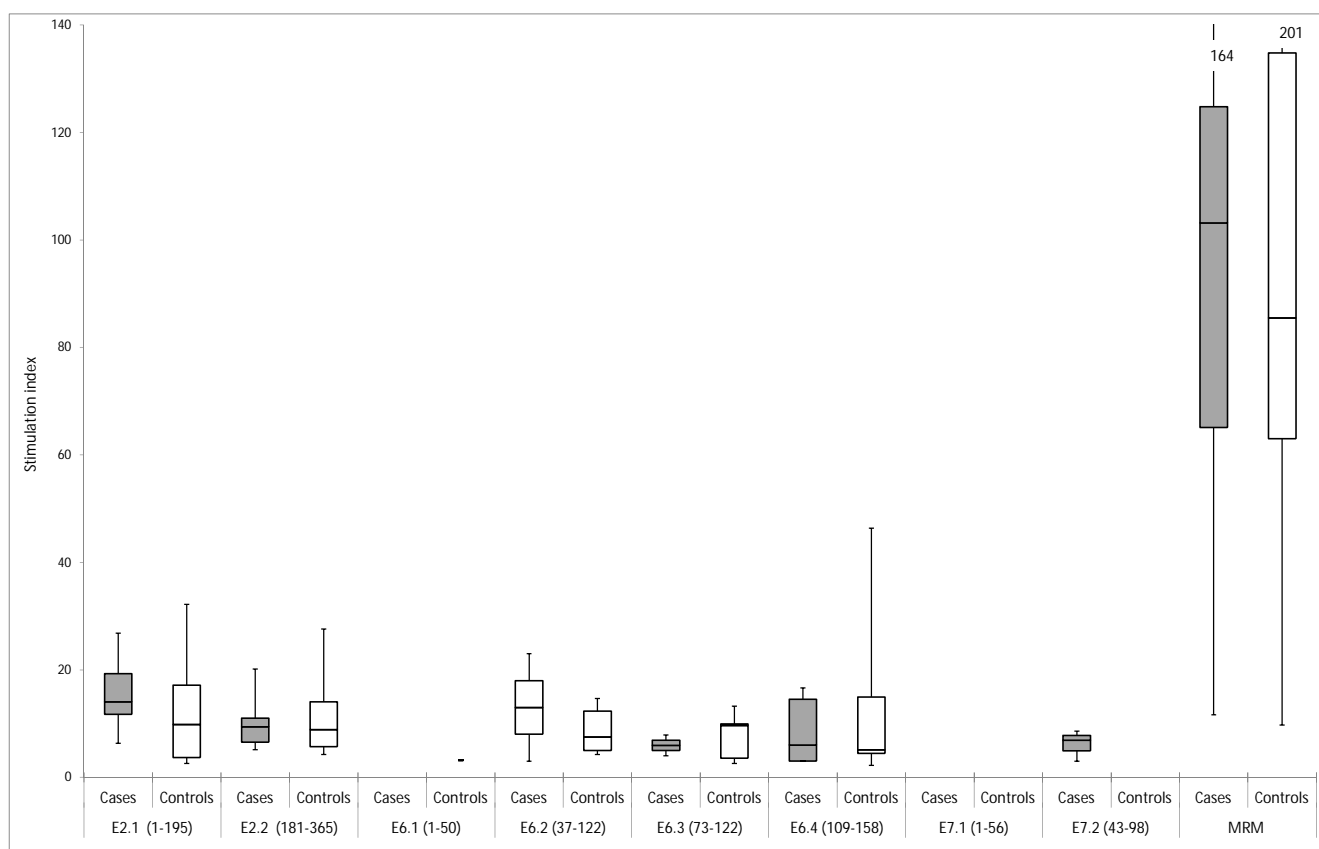

Supplement: Supplementary file 2 — 10.1186/s12967-015-0733-4 The oral HPV and HPV antibodies of the case-children from the baseline to 6 years (72 months). Oral HPV was tested at every time point, serum antibodies at the age of 1, 2, 6, 12, 24, and 36 months. Number in the box indicates the detected HPV type. Negative result is indicated by x and no sample by empty bar. [file 12967_2015_733_MOESM1_ESM.pdf]
