# Supplementary material for: Human papillomavirus 16-specific cell-mediated immunity in children born to mothers with incident cervical intraepithelial neoplasia (CIN) and to those constantly HPV negative
Source: J Transl Med. 2015 Nov 25;13:370. doi: 10.1186/s12967-015-0733-4 (PMC4659171; doi:10.1186/s12967-015-0733-4)
Supplement: Supplementary file 3 — 10.1186/s12967-015-0733-4 The oral HPV and HPV antibodies of the control-children from the baseline to 6 years (72 months). Oral HPV was tested at every time point, serum antibodies at the age of 1, 2, 6, 12, 24, and 36 months. Number in the box indicates the detected HPV type. Negative result is indicated by x and no sample by empty bar. [file 12967_2015_733_MOESM2_ESM.pdf]

		baseline	3 d	1 mo	2 mo	6 mo	12 mo	24 mo	36 mo	72 mo
ID1	oral HPV	x	x	x	x	x	x	x	x	
	Antibodies				x	x	x	x	x	
ID2	oral HPV	31	x	x	x	x	x	70	18	31
	Antibodies			x		x	x	16, 18	x	
ID3	oral HPV	31	6	33	x	x	x	x	x	31
	Antibodies				6	x	x	x	6	
ID4	oral HPV	x	x	x	x	x	x	x	x	
	Antibodies			16	x	x	x	x	6	
ID5	oral HPV	6	x	x	x	x	x	x	x	x
	Antibodies				x	x	6	6, 16, 18	x	
ID6	oral HPV	x	x	18	x	x	x	x	x	x
	Antibodies			x	x	6	6	6	6	
ID7	oral HPV	x	x	x	x	x	x	x		
	Antibodies			x	x	x	6	6		
ID8	oral HPV	16	6, 16	16	x	x	x	x	x	x
	Antibodies			x	x	6	6	x	x	
ID9	oral HPV	x	x	x		x				x
	Antibodies			6		x				
ID10	oral HPV	x	x	x		x	x	x	x	
	Antibodies			x	x	x	6			
